# Supplementary material for: The relationship between fibular head height and lower limb alignment deviation and severity after TKA for varus deformity knee osteoarthritis
Source: PLoS One. 2025 Jun 24;20(6):e0327168. doi: 10.1371/journal.pone.0327168 (PMC12186952; doi:10.1371/journal.pone.0327168)
Supplement: Attachment — Submitted filename: Response to Reviewers.docx [file pone.0327168.s003.docx]

**Dear Editor and Reviewer,**

**On behalf of my co-authors, we thank you very much for giving us an opportunity to revise our manuscript, and we also appreciate reviewers very much for their positive and constructive comments and suggestions on our manuscript entitled “The relationship between fibular head height and postoperative lower limb alignment deviation and severity after TKA for varus deformity knee osteoarthritis.” (Manuscript Number:  PONE-D-24-52473)**.

**Your comments were very helpful for revising and improving our paper, and provided valuable guidance to our research. We have studied your comments carefully and have made the appropriate corrections, which we hope will meet with approval. I hope you will consider that the revised content can be accepted for publication.**

**Thank you again for your valuable comments and suggestions. I look forward to hearing from you soon in due course.**

**Yours sincerely,**

**Hu Aixin.**

**Email: hu_aixin@163.com**

We really appreciate you for your carefulness and conscientiousness. Your suggestions are really valuable and helpful for revising and improving our paper. According to your suggestions, we have made the following revisions on this manuscript:

Reviewers' comments:

**Reviewer #1:**

**1. Title
It did not emphasize the highlights.**

**the term "Residual" is generic. It can be improved.**

*Response：Thank you very much for your advice. We revised the title from* “*Correlation between fibular head height and residual varus and valgus deformities after TKA for varus osteoarthritis” to “The relationship between fibular head height and postoperative lower limb alignment deviation and severity after TKA for varus deformity knee osteoarthritis” to better align with the results of the article. We plan to remove this term “Residual”, Post-operative residual varus angle and post-operative residual valgus angle are replaced with post-operative varus angle and post-operative valgus angle,*	*this makes it easier to understand. Thank you again for your constructive comments.*

**2.** **Abstract. It can be shortened.**

*Thank you very much for your advice.* *The abstract has been shortened without altering the original meaning，for details, please refer to the revised manuscript. Thank you again for your constructive comments.*

**3.The purpose is unclear. "Residual" need to be** **defined.**

*Response：**Thank you very much for your advice.* *The schematic diagram of all measurement indicators has been updated in the method section, and the postoperative varus angle and postoperative valgus angle have also been defined in the method section. Thank you again for your constructive comments.*

**4.In the methods, residual valgus was not specified clearly. It can be improved.**

*Response：T**hank you very much for your advice.* *This issue has already been explained in Question 3.* *Thank you again for your constructive comments.*

**5.The tibial rotation related with fibular height was not mentioned. Why not?**

*Response：Thank you very much for your advice.* *After reviewing the literature and combining the results of this study,* *we have some new findings as follows:* *The fibular head serves as the attachment point for several important ligaments and tendons, including the lateral collateral ligament, arcuate ligament, popliteofibular ligament, biceps femoris, and peroneus longus, among others. In addition, the popliteal tendon is indirectly connected to the fibular head through the popliteofibular ligament. Therefore, the position of the fibular head is closely related to the function of these ligaments and tendons.*

*Biomechanical studies have shown that, compared to the normal population, patients with varus knee osteoarthritis have higher peak activity levels of the peroneus longus and lower peak activity levels of the biceps femoris. On the first postoperative day following PFO surgery, the fibular head descends, and the activity level of the peroneus longus significantly decreases; by six months post-surgery, the activity level of the biceps femoris significantly increases and remains elevated. Theoretically, a decrease in fibular head height leads to laxity and reduced tension in the lateral collateral ligaments and tendons of the knee, which are crucial for maintaining knee stability.*

*The posterolateral ligament complex of the knee, including the lateral collateral ligament, arcuate ligament, popliteofibular ligament, and popliteal tendon, produces the greatest limiting force on tibial external rotation when the knee is flexed between 30° and 45°. When the knee is flexed between 0° and 30°, the lateral collateral ligament is the primary force limiting tibial external rotation; as the knee continues to flex, the popliteal tendon complex plays a more significant role. Therefore, the lateral soft tissues of the knee play an important role in limiting abnormal external rotation of the tibia.*

*In this study, it has been confirmed that in patients with varus knee osteoarthritis, the greater the degree of varus deformity, the lower the height of the fibular head. An elevated relative position of the fibular head may lead to decreased lateral soft tissue tension, resulting in knee instability in the horizontal position, manifesting as abnormal external rotation of the tibia. Previous studies have confirmed that the tibial external rotation angle in osteoarthritis patients is indeed abnormally increased, and with the progression of arthritis severity, the tibial external rotation angle also increases. Therefore, it can be hypothesized that fibular head height is a factor contributing to tibial torsion, which in turn leads to knee instability.* *Thank you again for your constructive comments.*

**6.** **Conclusion**

**It can be shortened. It is different from the main text.**

**The clinical relevance remains uncertain. It can be improved**

*Response：**Thank you very much for your valuable comments.* *The conclusion has been revised and adjusted in manuscript. Thank you again for your valuable suggestions.*

**7.** **Key words.**

**Some terms did not match in the Mesh Terms. It is suggested to check for proper terminology.**

*Response：Thank you very much for your valuable comments.* *After re-searching the PubMed website with Mesh Database, the keywords have been modified as follows,* *Keywords:* *fibula;* *knee osteoarthritis;* *Genu varus; Knee Replacement Arthroplasty. Thank you for your input.*

**8.** **Introduction**

**It can be shortened.**

*Response：**Thank you very much for your valuable comments.* *The introduction has been revised and adjusted in* *manuscript. The revised part of the introduction has been marked. Thank you again for your valuable suggestions.*

**9.** **The justification and contextualization were not stated clearly.**

*Response：Thank you for your insightful comment.* *After reviewing the relevant literature, the justification and background of this topic have been restated. You can* *see the details revise manuscript.* *Thank you again for your valuable inputs.*

**10.There are some unspecific terms like: Row 86:** **more common; Row 88: more pronounced; Row 97: varying degrees, Row 99:** **greater angle; Row 100:** **severe varus; Row 116:** **greater varus severity; row 117:** **significant differences**

*Response：Thank you very much for your valuable comments.* *Carefully read the sentences you pointed out in the introduction. Some of the sentences you pointed out do have ambiguity, and I will make the following changes.* *The term named “more common”* *is replaced by the term named “the incidence of varus deformity is higher than that of valgus deformity”; The term named “more pronounced”* *is replaced by the term named “more severe”; The term named “Varying degrees” is replaced by the term named “different degrees”;* *The term named “greater angle” is Replaced by the sentence named “it is more difficult to correct the lower limb force line in TKA surgery”; The term named “severe varus” is replaced by the term named “patients with severe varus deformity”; The term named “greater varus severity” is replaced by the term named “The greater the degree of varus”; The term named “significant differences” will be deleted. Thank you again for your valuable inputs.*
**11.** **It is lacking information about anatomic variation for fibular height and morphology, mainly between male and female It can be improved.**

*Response：Thank you very much for your comments. According to the results of the Mann-Whitney test in the revised manuscript, the fibular head in female patients is significantly closer to the lateral tibial plateau compared to male patients, indicating a statistically significant difference. Detailed information can be found in the statistical results section of the revised manuscript. Thank you again for your valuable feedback.*
**12.** **The classification for varus and valgus** **deformities were not mentioned. Why not?**

*Response：Thank you for your constructive comments. The classification of knee valgus and varus will be deformities given in the introduction,* *as follows:* *Based on the location of the deformity's apex, knee varus can be classified into tibial varus, femoral varus, or combined varus of both the femur and tibia.* *Furthermore, Thienpont and Parvizi recently proposed a new classification based on the location of the deformity, which includes intra-articular deformities, epiphyseal deformities, and diaphyseal deformities.* *Knee valgus deformity is classified into femoral valgus, tibial valgus, and combined valgus. Thank you again for your valuable inputs.*

**13.The different techniques for fibular height were not written. It can be improved.**

*Response：Thank you for your insightful comment.* *After reviewing the literature, the method for measuring fibular head height is as follows: the method for measuring the fibular head height in this study involves the vertical distance between the highest point of the fibular head and the horizontal tangent of the lateral tibial plateau of the knee joint. Since the tibial plateau is not perfectly flat, an alternative method involves measuring the vertical distance between the highest point of the fibular head and the lowest point of the tibial plateau along two horizontal tangents. Both of these methods are measured using X-ray images. Additionally, some studies have utilized three-dimensional CT to construct a model of the centroid of the fibular head, then measure the vertical distance between the centroid and the horizontal tangent of the lateral tibial plateau. Thank you again for your valuable inputs.*

**14.The hypothesis was not written. Why not?**

*Response：Thank you for your insightful comment.* *My previous hypothesis was presented in the introduction of the manuscript, but you may not have seen it. The hypothesis has now been revised due to the modification of the study title. Specifically, the hypothesis is as follows: In patients with varus deformity knee osteoarthritis, does the height of the fibular head affect the lower limb alignment after total knee arthroplasty (TKA)? Thank you again for your valuable inputs.*

**15.The purpose was not stated clearly. Row 132: What it means "significant difference"? statistically or clinically? It can be improved.**

*Response：Thank you for your insightful comment.* We have indeed observed clinically that in some patients with severe varus deformity and knee osteoarthritis, the fibular head is very close to the tibial plateau. Compared to healthy individuals, patients with varus deformity and knee osteoarthritis have a fibular head height that is closer to the tibial plateau, and this finding has been confirmed by relevant literature using data [2]. Therefore, in this study, we further classified the degree of varus deformity and used statistical methods to demonstrate that the fibular head height differs among patients with knee osteoarthritis at different degrees of varus deformity. *Thank you again for your valuable inputs.*

**16.** **Row 134"the residual varus or valgus were not determined with numbers. It can be improved.**

*Response：Thank you for your insightful comment. You pointed out the problem in the introduction. How to define the postoperative inversion angle and postoperative eversion Angle, I have re-described it in the method section. Thank you again for your valuable inputs.*

**17.** **Methods: The end points were not described clearly.**

*Response：Your insightful feedback is greatly appreciated.* *In the original manuscript, both the hip-knee-ankle (HKA) angle and the varus angle were used as indicators to assess the degree of knee varus. Therefore, in the methods section, the varus angle is now used as the indicator to assess the degree of knee varus, while the postoperative HKA angle is used as the indicator for evaluating lower limb alignment shifting after TKA. In the statistical analysis, I have used a varus angle of ≥10° and <10° as the grouping criteria, ensuring consistency throughout the manuscript. Thank you again for your valuable inputs.*

**18.The tibial torsion and knee rotational were not described. It is suggested to describe with more details**

*Response：Your comment has been very enlightening, and I sincerely appreciate it.*

*The fibular head serves as an attachment site for several important ligaments and tendons, including the lateral collateral ligament, arcuate ligament, popliteofibular ligament, biceps femoris, and fibularis longus. Additionally, the popliteal tendon indirectly connects to the fibular head through the popliteofibular ligament. Therefore, the position of the fibular head is closely related to the function of these ligaments and tendons.*

*Biomechanical studies have shown that, compared to healthy individuals, patients with varus-type knee osteoarthritis exhibit higher peak activity levels of the fibularis longus and lower peak activity levels of the biceps femoris. On the first postoperative day following PFO, the fibular head shifts downward, accompanied by a significant reduction in fibularis longus activity. By six months post-surgery, biceps femoris activity significantly increases and remains at a higher level. Theoretically, a reduction in fibular head height leads to a decrease in the tension and relaxation of the lateral knee ligaments and tendons, which are crucial for maintaining knee joint stability.*

*The posterolateral ligament complex of the knee, including the lateral collateral ligament, arcuate ligament, popliteofibular ligament, and popliteal tendon, exerts the greatest restraining force against tibial external rotation when the knee is flexed between 30°and 45°.From 0°to 30°of knee flexion, the lateral collateral ligament is the primary restraint against tibial external rotation. As knee flexion increases, the popliteal tendon complex becomes more prominent in restricting this motion. Therefore, the lateral soft tissues of the knee play a vital role in limiting abnormal external rotation of the tibia.*

*In patients with varus-type knee osteoarthritis, the elevated relative position of the fibular head may lead to decreased tension in the lateral soft tissues, resulting in knee instability in the horizontal plane, manifested as abnormal tibial external rotation. Previous studies have confirmed that the tibial external rotation angle is abnormally increased in osteoarthritis patients, with a further increase in external rotation as the severity of arthritis progresses.*

*Therefore, it can be hypothesized that fibular head height may be a contributing factor to tibial torsion, which in turn leads to knee joint instability.*

*I will add and modify the discussion section of the manuscript. Thank you again for your valuable inputs.*

**19．The sagittal view for varus and valgus deformity were not evaluated. Why not? It is suggested to add references for varus and valgus deformity, including residual varus and valgus.**

*Response：**Thank you very much for your valuable comments.* *I have added the sagittal diagram of the varus deformity, residual varus angle, valgus deformity and residual valgus angle as required，and all the pictures were improved in quality.* *Thank you again for your review.*

**20.** **The anatomic variation for fibular height and tibial anatomy, mainly between male female were not written. It is suggested to describe in more details.**

*Response：**Thank you very much for your comments.* *This question has been answered in question 11.* *Thank you again for your valuable feedback.*

**21.The technique used in the X-Ray was not written in details. How was determined the magnification and rotational problems?**

*Response：Thank you very much for your valuable comments.* *The X-ray technique used in this article has been described in the methods section.* *The details are as follows: This study used Wan dong DR equipment.* *The patient stood on both feet in front of the long box and received anteroposterior and posteroanterior radiographs in the standing position. The X-ray beam was centered on the knee joint, with the tube-to-tube distance of 10 feet, capable of encompassing both hip, knee, and ankle joints. The magnification rate of this method is typically around 5%.* *Thank you again for your suggestion.*

**22.** **Results**

**The statistical X clinical relevance were not described clearly. It can be improved.**

*Response：Your comment has been very enlightening, and I sincerely appreciate it.* *Specific details have been modified in the results section of the* *abstract. The details are as follows:* *The two groups were divided into those with an varus angle of ≥10° and <10°, and the height of the fibular head (8.1825±2.72505mm;.2234±2.68225mm; p=0.028<0.05) was statistically significant. In the unordered multinomial logistic regression analysis, with postoperative lower limb alignment (varus, valgus, neutral) as the dependent variable, height, weight, and body mass index (BMI) were found to be influencing factors for postoperative varus and valgus in patients with varus deformity and knee osteoarthritis. Among these, fibular head height was identified as an influencing factor for postoperative varus alignment. In the Pearson correlation analysis, fibular head height was positively correlated with the postoperative HKA angle (r = 0.212, p < 0.05), preoperative HKA angle was positively correlated with postoperative HKA angle (r = 0.393, p < 0.05), and the varus angle was positively correlated with the postoperative HKA angle (r = 0.171, p < 0.05).In the multiple linear regression analysis with postoperative HKA angle as the dependent variable, fibular head height and preoperative HKA angle were identified as significant influencing factors for the postoperative HKA angle (p < 0.05). Thank you again for your suggestion.*

**23.** **The morphotype of the participants were not written. Why not?**

*Response：Your comment has been very enlightening, and I sincerely appreciate it.* *The morphotype of the participants were written in general information.* *details are as follows:* *The patient demographics are as follows: 28 male and 105 female participants; age (67.59±7.03) years (range 53–84 years); height (159.38±6.23) cm (range 149–180 cm); weight (64.68±9.94) kg (range 42–95 kg); body mass index (25.4±3.38) kg/m² (range 17.5–35.8 kg/m²).Thank you again for your suggestion.*

**24.The accuracy of the measurements were not determined. Why not?**

*Response：Thank you very much for your valuable comments.* *In measuring the measurement index of this topic, each index is measured five times to take the average value and reduce the error generated by the measurement.* *This has been explained in the figure caption in the methodology section.* *Thank you again for your suggestion.*

**25.Discussion**

**The main findings were not correlated with clinical scenario. it can be improved.**

*Response：I greatly appreciate your insightful feedback* *The main statistical results combined with clinical discussion will be revised in the discussion section.* *I deeply value your thoughtful comments, thank you again.*

**26.** **The comparison among the technique used in this study could be more explored in this section. The clinical relevance remains unclear.**

*Response：Thank you very much for your valuable comments. In this study, the fibular head height was measured as the vertical distance between the superior edge of the fibular head and the horizontal tangent of the lateral tibial plateau from the sagittal plane. From the sagittal view, the fibular head is located on the posterolateral aspect of the tibia, and it can be clearly visualized on a true lateral X-ray of the knee, which meets the measurement requirements. X-ray, as a conventional imaging technique for knee osteoarthritis, is cost-effective and holds unique value from both diagnostic and patient care perspectives. However, knee X-rays present two-dimensional images, which cannot accurately assess the relative rotational relationship between the tibia and fibula. Additionally, some patients with varus deformity may develop fibular osteophyte formation due to the impact of knee osteoarthritis. These two factors contribute to measurement errors that cannot be completely eliminated in a two-dimensional plane. In a study by Ma Wenru and colleagues on the relationship between fibular head height and the onset and severity of varus knee osteoarthritis, the measurement method involved the distance between the superior edge of the fibular head and the horizontal tangent at the lowest point of the lateral tibial plateau. This method is similar to ours, but in healthy individuals, the distance between the fibular head and the lateral tibial plateau is already small, making this measurement method more difficult and prone to errors. Additionally, some studies have utilized three-dimensional CT to construct a fibular head centroid model and then measure the vertical distance between the centroid and the horizontal tangent of the lateral tibial plateau. The construction of the fibular head centroid model avoids the influence of osteophytes and the issue of relative rotation between the fibula and tibia. Moreover, it allows for more accurate measurement of the distance from the fibular head centroid to the lateral tibial plateau in three dimensions. I deeply value your thoughtful comments, thank you again.*

**27.** **Conclusion.**

**It can be shortened according with main findings and goal.**

**It is different from the abstract.**

*Response：Thank you very much for your valuable comments. According to my statistical data, the conclusion has been revised as follows:* *This study found that in patients with varus deformity knee osteoarthritis, the greater the degree of varus, the smaller the height of the fibular head; additionally, in female patients, the fibular head is closer to the lateral tibial plateau compared to male patients. In varus deformity knee osteoarthritis, the height of the fibular head is a contributing factor to the varus alignment of the lower limb's mechanical axis after TKA. The lower the fibular head, the more severe the varus alignment of the lower limb after TKA.Thank you again for your valuable suggestions.*

**Reviewer #2:**

1. **This manuscript contains numerous English errors that require correction before submission. For example, "steoarthritis" in line 129 should be corrected to "osteoarthritis," and "orrelation" in line 133 should be corrected to "correlation."**

*Response：Thank you very much for your valuable comments. Changes have been made in the original draft. Thank you again for your valuable suggestions.*

2. **In line 95, I am uncertain if "Osteotomy" is the most appropriate term for Total Knee Arthroplasty (TKA). Most publications utilize "bone resection" in this context.**

*Response：**Thank you very much for your valuable comments.* *After consulting a large number of relevant documents and discussing with my supervisor，We have decided to accept your suggestions. Thank you again for your valuable suggestions.*

3. **The presentation of results in the introduction section (lines 117-119: "We also found significant differences in the distance between the fibular head and the lateral plateau of the knee among patients with varying degrees of varus deformity") is unusual and should be relocated to the appropriate results section.**

*Response：Thank you very much for your valuable comments.* *After consideration, this sentence has been deleted.* *Thank you again for your valuable suggestions.*

**4.I am curious about the approximately equal distribution of patients between the cohorts (more and less than 10 degrees). Was this allocation randomized or accidental?**

*Response：Thank you very much for your valuable comments. First of all, according to the inclusion and exclusion criteria of my project, patients were randomly included. After measuring the fibula head height of all patients, literature was consulted to determine the indexing standard of varus Angle, which can be seen in the method part. Therefore, in the whole process of measuring fibula head height, we did not guide the distribution of cases in the two groups to be the same, perhaps because the sample size was not large enough. So this phenomenon is caused by chance. Thank you again for your valuable suggestions.*

**Response to editor：**

**Dear editor**

I would like to express my gratitude for your review. The corresponding author is currently listed as postdoctoral fellow Hu Aixin **(Email: hu_aixin@163.com)**, but I am the contact for the article. I would like to change the corresponding author to Hu Aixin. Once again, thank you very much for your comments and suggestions. And we hope that the revised manuscript can be accepted by PIOS ONE. If further revision is necessary, please contact me at: **hu_aixin@163.com**

Thank you and best regards.

Sincerely yours,

**Xun Qin**.

**Qinxun0930@foxmail.com**
