# Supplementary material for: The relationship between fibular head height and lower limb alignment deviation and severity after TKA for varus deformity knee osteoarthritis
Source: PLoS One. 2025 Jun 24;20(6):e0327168. doi: 10.1371/journal.pone.0327168 (PMC12186952; doi:10.1371/journal.pone.0327168)
Supplement: Attachment — Submitted filename: Response to Reviewers(2).docx [file pone.0327168.s004.docx]

**Dear Editor and Reviewer,**

**On behalf of my co-authors, we thank you very much for giving us an opportunity to revise our manuscript, and we also appreciate reviewers very much for their positive and constructive comments and suggestions on our manuscript entitled “The relationship between fibular head height and postoperative lower limb alignment deviation and severity after TKA for varus deformity knee osteoarthritis.” (Manuscript Number:  PONE-D-24-52473)**.

**Your comments were very helpful for revising and improving our paper, and provided valuable guidance to our research. We have studied your comments carefully and have made the appropriate corrections, which we hope will meet with approval. I hope you will consider that the revised content can be accepted for publication.**

**Thank you again for your valuable comments and suggestions. I look forward to hearing from you soon in due course.**

**Yours sincerely,**

**Hu Aixin.**

**Email: hu_aixin@163.com**

We really appreciate you for your carefulness and conscientiousness. Your suggestions are really valuable and helpful for revising and improving our paper. According to your suggestions, we have made the following revisions on this manuscript:

Reviewers' comments:

**Reviewer #3:**

**1. Lateral morphological changes of the knee joint occur with varus deformity of the knee joint and include upward migration of the fibular head. In other words, in patients with severe osteoarthritis of the knee, the** **fibular head is very close to the tibial plateau. This height of the fibular head, which contributes to tibial torsion and causes knee instability, was positively correlated with the postoperative HKA angle. Thus, patients with a low fibular head are more prone to lower extremity internal rotation after TKA. Therefore, patients with a low fibular head should undergo a less extensive tibial osteotomy, paying particular attention to medial soft tissue release and restoring as much lower extremity alignment as possible. In conclusion, it is recommended that patients with a low fibular head undergo proximal tibial osteotomy using the intraosseous alignment technique, which more effectively restores postoperative lower extremity alignment. Is this an acceptable conclusion? If so, please add a further concluding paragraph as short and concise as this one.**

*Response：Thank you very much for your advice.* *After reading your suggestions, I think it is necessary to add a short conclusion paragraph.* *Recently, I have reanalyzed the statistical results of this topic, especially in the results of multiple linear regression, and made corresponding revisions. First, I need to explain the term "* *fibular head height" in the text, adding the terms "low fibular head " and "high* *fibular head" In the text, fibular head height is denoted by H, and it has been defined in the section on measurement of observation indicators. A high fibular head indicates a smaller vertical distance between the tibial tuberosity and the lateral platform of the knee joint, while a low tibial tuberosity indicates the opposite.*

*After reviewing relevant materials and combining them with the results of this study, some conclusions in your suggestions need to be revised. For example, "Therefore, patients with a low fibular head should undergo a less extensive tibial osteotomy, paying particular attention to medial soft tissue release and restoring as much lower extremity alignment as possible.* *In conclusion, it is recommended that patients with a low fibular head undergo proximal tibial osteotomy using the intraosseous alignment technique, which more effectively restores postoperative lower extremity alignment. " has revised to "Therefore, patients with a high fibular head (small H value) should undergo a less extensive tibial osteotomy, paying particular attention to medial soft tissue release and restoring as much lower extremity alignment as possible.* *In conclusion, it is recommended that patients with a high fibular head undergo proximal tibial osteotomy , which more effectively restores postoperative lower extremity alignment. "* *This modification will be more in line with the conclusion of this topic.* *A brief conclusion paragraph has been added to the end of the discussion, as follows:* *The lateral morphological changes in the knee joint occur due to varus deformity of the knee, including upward displacement of the fibular head. In other words, in patients with severe varus deformity of the knee, the fibular head is very close to the tibial plateau. The height of the fibular head is positively correlated with the postoperative HKA angle; low fibular head height （High-positioned fibular head）can lead to knee instability. Therefore, patients with varus deformity of the knee and a higher fibular head (low fibular head height) are more likely to develop lower limb varus deformity after total knee arthroplasty. For patients with varus deformity of the knee and a higher fibular head, a smaller range of tibial osteotomy should be performed, paying particular attention to medial soft tissue release ,do not perform extensive release, aiming to restore the alignment of the lower limb as much as possible. Finally, in clinical practice, it is necessary to routinely measure the height of the fibular head in patients with varus deformity of the knee and osteoarthritis.* *Thank you again for your constructive comments.*

**2. Furthermore, the recent concept of kinematic alignment allows for varus deformity after TKA. If this is the case, the above caution would no longer be necessary, but I thought the paper would be better if the author's thoughts on this point were also added somewhere.**

*Response：I would like to express my gratitude again for your constructive observations. Regarding the kinematics of TKA alignment, I will elaborate on this in the third sub-point of the discussion section. Specifically as follows: In recent years, kinematic alignment has been associated with postoperative varus malalignment of the lower limb following TKA. However, the authors believe that traditional mechanical alignment still retains its significance. The effectiveness of traditional mechanical alignment in TKA is widely recognized, with generally favorable outcomes and a prosthesis survival rate exceeding 90% at 15 years. Kinematic alignment advocates for preserving the native anatomy of the knee by avoiding ligament release and complete correction of preoperative varus, valgus, or flexion deformities. Although kinematic alignment allows for a degree of postoperative varus alignment, in patients with severe preoperative varus osteoarthritis, excessive tightness of the medial soft tissues can significantly compromise postoperative limb alignment. Technological advancements, including patient-specific cutting guides, computer-assisted navigation systems, and orthopedic robotics, have been introduced to support kinematic alignment in TKA, showing clear advantages over traditional mechanical techniques. However, in resource-limited settings, traditional mechanical alignment remains the predominant approach. Therefore, the authors believe that the value of mechanical alignment should not be underestimated.* *Thank you again for your constructive comments.*

**Reviewer #4:**

**1.First, I have difficulty in agreeing that the height of the fibular head is a contributing factor in arthritis and knee coronal plane deformity. The height can affect the stability surely by the contribution of the fibular head the attachment of the lateral stabilizing structures but the femorotibial joint alignment is affected by the collapse of the femoral or tibial condyles as mentioned by the authors in the introduction. The fibula here is extraarticular structure and the fibular height before or after surgery and its relation to the coronal plane alignment is merely observational and secondary to the original alignment as long as there is no lateral major instability.**

*Response：I would like to express my gratitude again for your constructive observations.* *First of all, this topic is a correlation study. For the height of the fibular head is a contributing factor to arthritis and coronal deformity of the knee joint, I will change it to a relevant factor rather than a contributing factor in the discussion section of this topic.* *In the second point of the discussion, instead of stating that fibular head height contributes to tibial torsion and subsequently leads to knee instability, it can be rephrased as: In patients with varus knee osteoarthritis, a high-positioned fibular head (low fibular head height) is a factor associated with knee joint instability. Thanks again for your helpful and constructive comments.*

**2.Second. the choice of the cutoff value determined as 10 degrees of varus aligment and its use in statistical comparison.**

*Response：Thank you very much for your advice.* *The group is divided based on an internal angle of 10° as the cutoff value because this threshold is too small, leading to significant measurement errors. This also aligns with the grading criteria for the degree of varus.* *This also aligns with the grading criteria for the degree of varus.* *Thank you again for your constructive comments.*

**3.Third, the conclusions and recommendations from this study should be revised as for example. The finding that patients with a lower fibular head were more likely to experience postoperative varus alignment of the lower limb after total knee arthroplasty (TKA) and therefore, it is essential to routinely measure fibular head height in patients with varus deformity knee osteoarthritis in clinical practice. The recommendation of the choice of intramedullary or extramedullary alignment method, the authors declare that it is recommended to use the intramedullary alignment method during surgery for proximal tibial bone resection. This is controversial as well.**

*Response：Thank you very much for your advice. According to your suggestion, I have made some changes in the text. As detailed below:* *This study found that in patients with varus deformity knee osteoarthritis，a greater degree of varus was associated with a lower fibular head height. Additionally，the fibular head in female patients was positioned closer to the lateral tibial plateau compared to male patients. In varus deformity knee osteoarthritis，fibular head height is a risk factor for postoperative lower limb varus alignment following total knee arthroplasty (TKA). Patients with a higher-positioned fibular head (lower fibular head height) are more likely to develop postoperative varus malalignment after TKA. Therefore，routine measurement of fibular head height is warranted in clinical practice for patients with varus deformity knee osteoarthritis.*

*About the recommendation of the choice of intramedullary or extramedullary alignment method，It has been discussed with other authors and it has been decided to delete it.* *The lower the height of the fibular head (high fibular head), the greater the degree of valgus of the lower limb after TKA, which has been explained in question 1.* *Thank you again for your constructive comments.*

**Response to editor：**

**Dear editor**

I would like to express my gratitude for your review. The corresponding author is currently listed as postdoctoral fellow Hu Aixin **(Email: hu_aixin@163.com)**, but I am the contact for the article. I would like to change the corresponding author to Hu Aixin. Once again, thank you very much for your comments and suggestions. And we hope that the revised manuscript can be accepted by PIOS ONE. If further revision is necessary, please contact me at: **hu_aixin@163.com**

Thank you and best regards.

Sincerely yours,

**Xun Qin**.

**Qinxun0930@foxmail.com**
